# Protective factors of social-emotional well-being in preschool-aged children: a comparative descriptive study

**DOI:** 10.1186/s12887-026-07422-0

**Published:** 2026-08-06

**Authors:** Mariska Elizabeth Oosthuizen-van Tonder, Suegnét Scholtz, Emmerentia du Plessis

**Affiliations:** NuMIQ Research Focus Area, Faculty of Health Sciences, North-West University, Potchefstroom, South Africa

**Keywords:** Early childhood development, Mental health promotion, Preschool children, Protective factors, Social-emotional well-being

## Abstract

**Background:**

Internationally, school-based psychosocial services are increasingly integrated into primary education. However, in South Africa, such services remain in the planning phase and largely exclude preschoolers, for whom clear service pathways and universally accessible mental health services are absent. Given that early social-emotional competence underpins later learning, understanding these needs is vital. This study offers an initial empirical contribution towards identifying preschool children’s social-emotional well-being needs, which may inform programme developers and policymakers in designing targeted mental health services. The study explored this need by comparing protective factors of social-emotional well-being (initiative, self-regulation, and attachment/relationship) with preschoolers’ demographics.

**Methods:**

A quantitative comparative descriptive design was used to collect data from a convenience sample of middle-class parents of children aged 3–5 years (*n* = 150) in 2023, shortly after COVID-19. The Devereux Early Childhood Assessment for Preschoolers (DECA-P2) behavioural rating scale was reportedly used for the first time in a South African context. Descriptive statistics, independent t-test, and one-way analysis of variance (ANOVA) were conducted using SPSS 29.0.

**Results:**

The mean of protective factors (3.79) and subscales, ‘initiative’ (3.81) and ‘self-regulation’ (3.44), fell within the ‘typical’ range, indicating normative functioning without pronounced strengths or deficits. The mean score of ‘behavioural concerns’ (2.08) was also ‘typical’. However, ‘attachment/relationship’ (4.13) emerged as an ‘area of concern’, indicating that children with lower scores may benefit from additional support to strengthen their social-emotional connections.

**Conclusion:**

While preschoolers from stable, middle-class families typically demonstrated resilience across protective factors, ‘attachment/relationship’ emerged as a domain where additional support could promote stronger socio-emotional well-being. Upcoming programmes should foster stronger ‘attachment/relationship’, enhance parental support, and ensure access to structured services tailored to pre-school-aged children in South Africa. This study underscores the importance of accessible mental health services tailored for preschoolers in South Africa. It provides a starting point for understanding the social-emotional needs of preschoolers in middle-class contexts within the North West province. It informs strength-based interventions that build on resilience while addressing areas for support. The research may inform promotive programme design and support evidence-based early childhood policies.

## Introduction

Mental health services for children are increasingly recognised as vital in health and educational settings, yet services for preschool-aged children remain limited, especially in low- and middle-income countries [1]. Early childhood is a crucial period for emotional and social development [2], and unmet needs during this time can have long-term consequences [3, 4]. In South Africa, recent efforts such as the 2024 Psychosocial Support Services programme [5, 6] show promise, but some regions in the North West province still face significant service gaps. Broader literature suggests that the COVID-19 pandemic may have intensified mental health challenges [7, 8], highlighting a need for accessible services for preschool children. Preschool-aged children in low- and middle-income regions, particularly in South Africa’s North West province, lack accessible and structured mental health services despite the critical importance of social-emotional development as a core domain of early childhood development. This study used the DECA-P2 assessment tool to explore the social-emotional well-being (SEWB) of preschoolers from low- to middle-income families in the North West province. It provides a baseline for identifying service needs and informs strength-based interventions that build on resilience while addressing areas for support.

Although the value of mental health services within health and education settings is increasingly acknowledged worldwide [9], mental health care services for preschool-aged children (3–5 years) remain insufficient, especially across low- and middle-income countries. The first five years of a child’s life constitute a critical developmental window [10], laying the groundwork for a child’s holistic development, which encompasses their physical, cognitive, social, and emotional growth [11].

Mental health challenges, a common non-communicable disease, are the third-leading cause of overall disease worldwide [12]. According to the World Health Organisation (WHO), well-being represents a comprehensive state of physical, mental, and social functioning, rather than merely the absence of impairment. Within this framework, social-emotional well-being (SEWB) is a critical component of children’s early developmental trajectory, serving as a protective factor that underpins overall mental health. Emotional well-being generally reflects a sense of happiness and security, whereas social well-being generally entails the ability to form and maintain positive relationships [13].

In 2018, the WHO committed to reducing premature mortality from non-communicable diseases by one-third by 2030 through mental health promotion, prevention, and treatment, an initiative aligned with Sustainable Development Goal (SDG) 3.4. By prioritising the reduction of premature mortality through mental health promotion, SDG 3.4 highlights the need to address mental health early in life, recognising that childhood interventions lay the foundation for lifelong well-being and resilience [14, 15].

Globally, psychosocial services in schools are becoming a standard part of primary education. In South Africa, the Department of Basic Education launched a national Psychosocial Support Services programme in 2024, piloted in two provinces, Gauteng and KwaZulu-Natal. The programme focuses on the role of educators to create supportive learning environments that enhance SEWB among school-aged children [16]. At-risk children are identified and supported through a referral system to strengthen their resilience and lessen the possibility of the development of mental health challenges [17].

Although this initiative is expected to yield positive outcomes, there remains a broad consensus that child and adolescent mental health services in low- and middle-income countries, including South Africa, require urgent and sustained attention [18, 19]. The need for accessible services in the North West province is particularly acute, as the region has been identified as having minimal mental healthcare infrastructure [20, 21]. Assessing the current SEWB of children in this context is therefore an urgent priority that cannot be deferred until systemic service delivery challenges are resolved.

South Africa has made progress towards achieving SDG 3 through the anticipated introduction of National Health Insurance (NHI), promising to deliver health services for all. However, the health system remains inequitable. In the aftermath of the COVID-19 pandemic, the country faces escalated mental health needs, with even greater resource shortages and limited access to healthcare [7, 8, 22–25]. Numerous socio-economic factors threaten the SEWB of preschool children [22]. As a developing country, SEWB continues to receive insufficient attention, and the limited availability and accessibility of mental health services place preschool children at risk for developing mental health challenges later in life [26–28].

There remains a significant service gap due to the lack of clear service pathways and universally accessible mental health services for preschoolers [14]. Without an understanding of children’s current social-emotional well-being, it is difficult to design responsive promotive interventions that meet their needs. By establishing a clearer understanding of children’s SEWB, stakeholders may be better positioned to design inclusive, broadly accessible mental health promotional programmes embedded in early childhood educational centres and primary healthcare clinics, aiming to support environments that promote resilience and emotional competence among children, regardless of their identified risk [29].

The researchers adopt a strength-based approach to SEWB, focusing on the capability and the development of protective factors (namely, initiative; self-regulation; and attachment or relationships) while reducing behavioural concerns as indicators of overall SEWB [30]. This study identifies protective factors of SEWB and compares them with demographic characteristics that may influence the SEWB of preschool children. This study offers a baseline for stakeholders, identifying resilience and promotive support needs in preschool children (aged 3–5 years) in the North West province. The findings serve as a contextual starting point to inform future service development and policy implementation, while recognising the exploratory nature of the sample.

## Method

### Study design and sampling

The relationships between the variables were determined using a non-experimental comparative descriptive research design, aiming to identify protective factors, areas of concern, and possible demographic characteristics that may influence the SEWB of preschool children. A convenience sample (*n* = 150) was taken from eight different schools in the North West province.

These eight schools were identified through the Education Management Information System (EMIS). The schools are registered with the Department of Education as a primary comprehensive school. According to this system, a ‘primary comprehensive school’ refers to an institution that provides both foundation and intermediate phase education and often includes an Early Childhood Development (ECD) centre for children aged 3–5 years. If the school, on the EMIS, did not have an attached public preschool, the closest independent preschool was chosen.

Stratified sampling was applied to ensure representation across four districts (Dr Kenneth Kaunda, Bojanala Platinum, Dr Ruth Segomotsi, Ngaka Modiri Molema). Within each district, two ECD centres were selected to reflect variation in preschool type (government-attached and independent), serving as proxies for socio-economic status (SES) and geographical context (urban versus rural). This stratification captured diversity in service provision and family background, contextualising the need for accessible mental health services for preschool-aged children. Parents were then recruited through convenience sampling, which limits generalisability but provides valuable exploratory insights. Permission to conduct the study was granted by the North West Department of Education, and ethical approval was obtained from the NWU-HREC (reference number NWU-00008-22-A1).

### Study context

The study was conducted in the North West province of South Africa, a region characterised by marked socio-economic disparities and limited access to structured mental health services for preschool children. To ensure representation across diverse settings, all four districts were purposively selected: Dr Kenneth Kaunda, Bojanala Platinum, Dr Ruth Segomotsi, and Ngaka Modiri Molema. These districts were chosen to reflect variation in geographical setting (urban vs. rural), SES, and access to educational and health infrastructure. Dr Kenneth Kaunda and the Bojanala Platinum are predominantly urban, whereas Ngaka Modiri Molema and Dr Ruth Segomotsi are largely rural. Schools were identified through the EMIS and included both independent and public schools registered with the Department of Education. These schools represent the most common preschool provision in the province, enrolling children aged 3–5 years (Grade RRR-R programmes) prior to formal schooling. The schools varied in type, including preschools operating with limited resources and government schools serving middle-income families. This diversity in school type and district context provided a basis for identifying social-emotional well-being needs across different service environments. However, the sample largely reflected stable, middle-class families (married, educated, homeowners, majority Caucasian), which should be acknowledged when interpreting findings.

### Participant characteristics

SEWB in preschool and early childhood is shaped by broader contextual conditions, structural inequalities, and interactions between family and school, rather than by individual factors alone. Ethnicity was included as a demographic variable because research shows that children from ethnic minority backgrounds have disproportionately high unmet mental health needs, largely due to limited access to resources and services [31]. Similarly, other social determinants of health, such as parental education, geographical location, number of siblings, and gender, are contextual factors that may influence children’s SEWB [32–34].

**Fig. 1:**
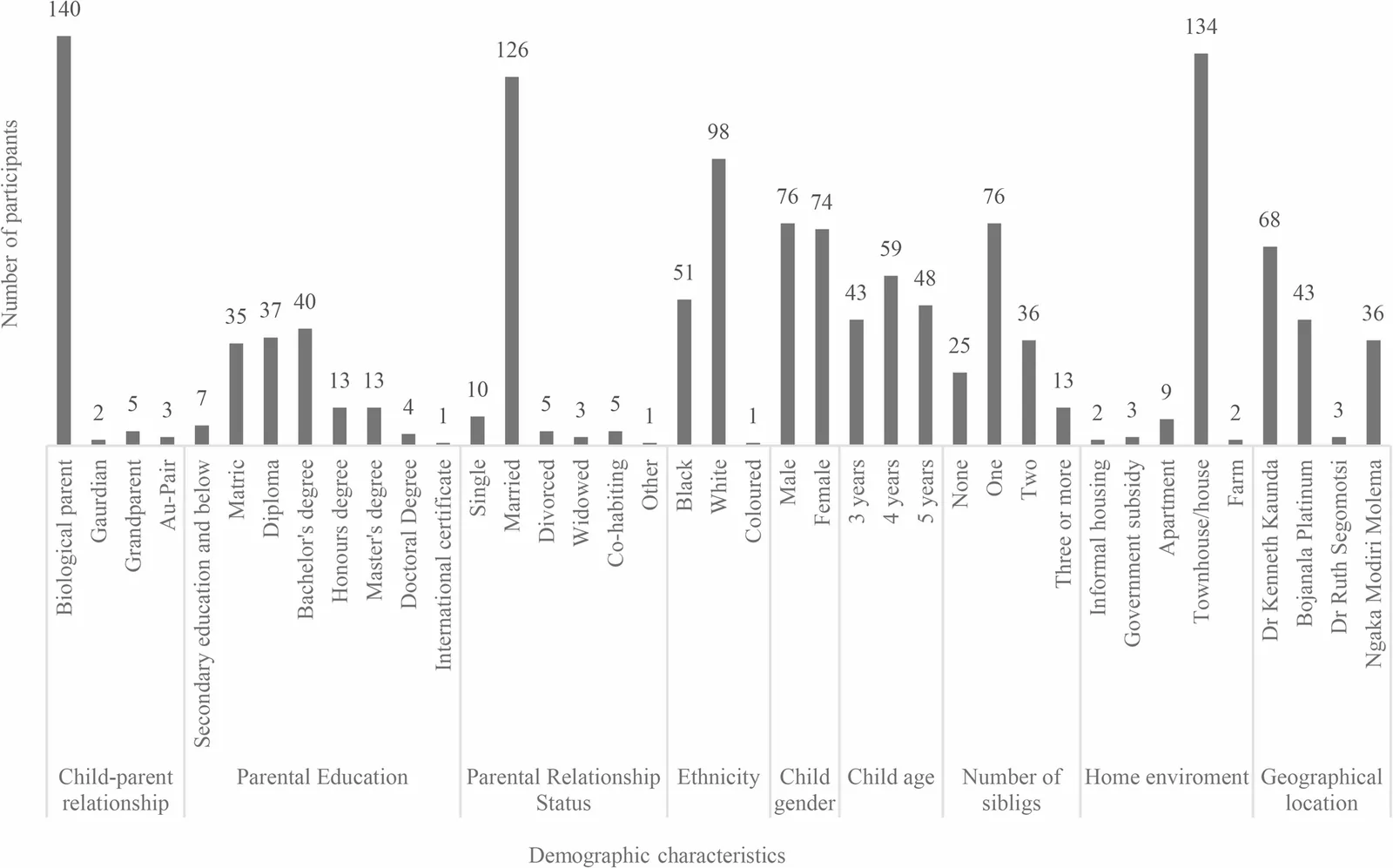
Demographic characteristics of research participants (*N* = 150)

The majority of the participants were biological parents (*n* = 140, 93.3%), Caucasian (*n* = 98, 65.5%), married (*n* = 126, 84%), with educational levels ranging from a national diploma (matric) to a bachelor’s degree (*n* = 112, 74.7%), and staying in a house or townhouse (*n* = 134, 89.3%). Participants’ children had the following characteristics: Half of the children had one sibling (*n* = 76, 50.7%), while the remaining half were divided equally: one quarter had two siblings (*n* = 36, 24%), while others had no siblings (*n* = 25, 16.7%), only a small proportion of the children had two or more siblings (*n* = 13, 8.6%). The children’s gender was equally distributed with 50.7% boys (*n* = 76) and 49.3% girls (*n* = 74). A total of 28.6% of the children were three years old (*n* = 43), 39.3% were four years old (*n* = 59), and 32.1% were five years old (*n* = 48). Participant characteristics are presented in Fig. 1.

### Data collection

Parents or caregivers of preschool-age children were recruited through posters displayed at participating schools. Recruitment materials included a QR code linking participants directly to the online informed consent form and the DECA-P2 questionnaire (English version). Data were gathered at designated school sites, where computers or tablets were made available to participants to ensure access for everyone.

To participate, parents or caregivers were required to have a child between the ages of three and five years (before their sixth birthday) who was in their care for at least three days per week. Children diagnosed with a mental health disorder were excluded from participation to maintain the study’s strength-based focus on protective factors in typically developing preschoolers. No additional inclusion criteria were applied. All data were gathered confidentially, with assistance from school gatekeepers (e.g., school secretaries or principals) who helped provide access to participants.

### Devereux early childhood assessment for preschoolers

Data were collected using the Devereux Early Childhood Assessment for Preschoolers (DECA-P2), applied for the first time in a South African population. The DECA-P2 is a parent-completed behaviour rating scale designed to assess within-child protective factors central to social-emotional well-being and resilience, as well as behavioural concerns in preschool children. Classified as a Level One assessment tool [35], it meets high methodological standards and has strong empirical support. The instrument demonstrates robust normative data [30, 36], high internal consistency of α = 0.92 for parents-raters [37], and validated construct validity [36, 38–40] and content validity [41]. It also aligns statistically with established tools such as the Conners Early Childhood Scale [38] and the Preschool Behavioral and Emotional Rating Scale [39], confirming its reliability across diverse contexts, making it a reliable choice for identifying SEWB in preschoolers [42].

DECA-P2 includes three protective factor scales: *initiative*: ‘the child’s ability to use independent thought and action to meet their needs’; *self-regulation*: ‘the child’s ability to express emotions and manage behaviours in healthy ways’; and *attachment or relationships*: ‘the child’s ability to promote and maintain mutual, positive connections with other children and adults’. Raw scores are converted into T-scores and percentile ranks using the DECA-P2 norms Table (30). The DECA-P2 used in this study was approved and supplied by the Kaplan Early Learning Company.

Reliability was assessed using Cronbach’s alpha coefficients, a widely recognised indicator of internal consistency [43–45]. Reported coefficients ranged from 0.68 to 0.92, demonstrating acceptable to excellent reliability [46]. Specifically, the protective factors scale yielded α = 0.92, while subscales showed values of 0.84 (self-regulation), 0.86 (initiative), and 0.79 (attachment/relationships).

### Data analysis

Data analysis was conducted using SPSS version 29.0. Independent t-tests and one-way ANOVA were performed to examine group differences, with statistical significance set at *p* ≤ 0.05. Because the sample was non-random, effect sizes were calculated in addition to these tests, using Cohen’s d, interpreted as small = 0.2, medium = 0.5, and large = 0.8 [47]. There were no missing items in the data set. One-way ANOVA was not conducted for the Dr Ruth Segomotsi geographic subgroup due to the small number of participants (*n* = 3) from this district. The reduced sample size did not satisfy the minimum requirements for stable variance estimation, and any comparisons would likely have yielded statistically unreliable and non-informative results [48].

The DECA-P2 ratings are based on the child’s observed behaviour over the past four weeks, with parents or caregivers completing the 38 Likert scale items (0 = never to 4 = very frequently). Raw scores were summed for each scale, and T-scores and percentile ranks were determined using the norms table from LeBuffe and Naglieri [30]. For the protective factor scales, high T-scores (≥ 60) were classified as ‘strengths’, scores between 41 and 59 as ‘typical’, and scores ≤ 40 as an ‘area of need’. For the behavioural concerns sub-scale, high T-scores (≥ 60), while scores below 60 were considered typical. The behavioural concerns sub-scale has no strengths.

A statistician was consulted to ensure accuracy, and findings were grouped according to the relevant SEWB components: protective factors (initiative, self-regulation, and attachment or relationship) and behavioural concerns. Differences between demographic variables and SEWB components were then analysed.

## Results

### Percentile ranks of overall T-scores

Percentile ranks indicate the number of children who earned the same or a lower score. Indicating that the participants scored 34 on the percentile rank, which means that 34% of children in the standardisation sample earned the same or a lower score. Each DECA-P2 T-score is a standard score that is set to have a mean of 50 and a standard deviation of 10. Like the percentile ranks, T-scores are based on the ratings received by the children in the standardisation sample [30]. Overall, T-scores were calculated by taking the averages of the questions within the specific protective factor measured. These scores can fall within specific categories classified as an ‘area of concern’ or as ‘typical’. Children who obtain scores within the ‘typical’ range demonstrate resilience and normative functioning and may still benefit from participation in a promotion programme to further strengthen resilience and protective buffering against potential stressors and adversities. Action is needed for scores within the ‘areas of concern,’ ranges, as children might need a targeted prevention plan to address the ‘area of concern’. As set out in Table 1, the participants (*N* = 150) in the North West had the following T-scores and percentile ranks.

**Table 1:** SEWB in children percentile rankings of overall T-scores

SEWB components	Total item rating (*N* = 150)	T-score	Percentile rank	Description of each scale
1. Protective factors	128	41	18	Typical
1.1. Initiative	25	46	34	Typical
1.2. Self-regulation	22	44	27	Typical
1.3. Attachment or relationship	27	38	12	Area of concern
2. Behavioural concerns	11	50	50	Typical

### Independent t-tests

As presented in Table 2, Independent t-tests were performed to determine if there were differences between the means of two unrelated groups [43]. Two sets of t-tests were performed, one on the different scales and child gender, and one on the different scales and ethnicity. Interpretation of t-tests is based on effect size as guideline values [49]. Note that p-values are reported for completeness’s sake but were not interpreted since a convenience sample was used.

**Table 2:** Independent t-test for parent ethnicity and child gender

	PARENT ETHNICITY						CHILD GENDER					
SEWB components		N	Mean	Std. Deviation	*p-value	Effect size		N	Mean	Std. Deviation	*p-value	Effect size
1. Protective factors	African	51	3.72	0.50	0.30	0.18	Boys	76	3.71	0.47	0.04	0.33
	Caucasian	98	3.81	0.47			Girls	74	3.88	0.50		
1.1. Initiative	African	51	3.74	0.68	0.55	0.10	Boys	76	3.69	0.61	0.02	**0.38**
	Caucasian	98	3.81	0.59			Girls	74	3.92	0.62		
1.2. Self-regulation	African	51	3.41	0.57	0.74	0.06	Boys	76	3.38	0.56	0.15	0.23
	Caucasian	98	3.45	0.57			Girls	74	3.51	0.59		
1.3. Attachment or relationship	African	51	4.01	0.51	0.06	0.32	Boys	76	4.07	0.52	0.14	0.24
	Caucasian	98	4.18	0.52			Girls	74	4.20	0.53		
2. Behavioural concerns	African	51	2.06	0.41	0.70	0.06	Boys	76	2.16	0.49	0.02	0.35
	Caucasian	98	2.09	0.49			Girls	74	1.99	0.42		

*p-values are reported but not analysed due to convenience sampling. The parental ethnicity group ‘coloured’ was not tabulated in Table 2 due to the small representative sample of < 1% in this ethnicity group

### One-way ANOVA

As presented in Table 3, one-way ANOVA is a method for assessing the significance of two means between two or more groups with one dependent and one independent variable by comparing variability within groups and between groups [50, 51]. ANOVA was performed on child age, geographical area, number of siblings, and parental education.

**Table 3:** One-way ANOVA results of child age, geographical area, number of siblings, parental education, and SEWB component

CHILD AGE								GEOGRAPHICAL AREA								
Age	*N*	Mean	SD	ANOVA *p*-value	Welch *p*-value	Effect size		Area	*N*	Mean	SD	ANOVA *p*-value	Welch *p*-value	Effect size		
						3Y	4Y							Dr KKDM	Bojanala	
Protective factors																
3Y	43	3.77	0.53	0.73	0.69			Dr KKDM	68	3.86	0.45	0.35	0.29			
4Y	59	3.76	0.53			0.01		Bojanala	43	3.77	0.55			0.16		
5Y	48	3.83	0.41			0.13	0.13	Ngaka	36	3.73	0.38			0.29	0.07	
Total	150	3.79	0.49					Total	147	3.80	0.47					
Initiative																
3Y	43	3.83	0.64	0.82	0.80			Dr KKDM	68	3.90	0.56	0.23	0.20			
4Y	59	3.82	0.66			0.03		Bojanala	43	3.77	0.69			0.19		
5Y	48	3.75	0.57			0.17	0.20	Ngaka	36	3.70	0.57			0.35	0.10	
Total	150	3.80	0.62					Total	147	3.81	0.61					
Self-regulation																
3Y	43	3.37	0.61	0.24	0.20			Dr KKDM	68	3.47	0.57	0.72	0.66			
4Y	59	3.39	0.60			0.03		Bojanala	43	3.46	0.62			0.01		
5Y	48	3.55	0.50			0.29	0.27	Ngaka	36	3.38	0.47			0.15	0.14	
Total	150	3.44	0.58					Total	147	3.44	0.56					
Attachment or relationship																
3Y	43	4.10	0.57	0.52	0.45			Dr KKDM	68	4.20	0.46	0.34	0.36			
4Y	59	4.08	0.57			0.03		Bojanala	43	4.07	0.61			0.22		
5Y	48	4.19	0.42			0.17	0.20	Ngaka	36	4.10	0.42			0.21	0.06	
Total	150	4.12	0.53					Total	147	4.14	0.50					
Behavioural Concerns																
3Y	43	2.07	0.48	0.99	0.99			Dr KKDM	68	2.14	0.50	0.10	0.08			
4Y	59	2.07	0.45			0.01		Bojanala	43	2.09	0.43			0.09		
5Y	48	2.07	0.47			0.02	0.00	Ngaka	36	1.93	0.41			0.40	0.37	
Total	150	2.07	0.46					Total	147	2.07	0.46					

NUMBER OF SIBLINGS								PARENTAL EDUCATION								
No	N	Mean	SD	ANOVA p-value	Welch *p*-value	Effect Size		Education	N	Mean	SD	ANOVA *p*-value	Welch *p*-value	Effect Size		
						None	One							Primary-Matric	Diploma	Bachelor’ degree
Attachment or relationship																
None	25	4.01	0.70	0.42	0.53			Primary-Matric	41	3.93	0.60	0.01	0.02			
1	76	4.17	0.41			0.23		Diploma	37	4.15	0.52			0.36		
2/<	49	4.12	0.58			0.16	0.08	Bachelor’s degree	40	4.29	0.39			0.60	0.29	
								Postgraduate	30	4.19	0.46			0.43	0.08	0.23
Total	150	4.13	0.53					Total	148	4.14	0.51					
Behavioural Concerns																
None	25	2.03	0.51	0.87	0.89			Primary-Matric	41	2.10	0.52	0.64	0.65			
1	76	2.08	0.46			0.11		Diploma	37	2.03	0.43			0.13		
2/<	49	2.08	0.43			0.09	0.01	Bachelor’s degree	40	2.03	0.41			0.15	0.02	
								Postgraduate	30	2.15	0.51			0.10	0.23	0.25
Total	150	2.07	0.46					Total	148	2.07	0.47					

NUMBER OF SIBLINGS								PARENTAL EDUCATION								
No	N	Mean	SD	ANOVA *p*- value	Welch *p*-value	Effect Size		Education	N	Mean	SD	ANOVA *p*-value	Welch *p*-value	Effect Size		
						None	One							Primary-Matric	Diploma	Bachelor’s degree
Protective factors																
None	25	3.63	0.66	0.22	0.41			Primary-Matric	41	3.69	0.59	0.38	0.46			
1	76	3.81	0.42			0.27		Diploma	37	3.84	0.45			0.26		
2/<	49	3.83	0.48			0.30	0.04	Bachelor’s degree	40	3.86	0.40			0.29	0.04	
								Post-graduate	30	3.79	0.46			0.17	0.12	0.16
Total	150	3.79	0.49					Total	148	3.80	0.48					
Initiative																
None	25	3.65	0.77	0.22	0.28			Primary-Matric	41	3.74	0.67	0.77	0.78			
1	76	3.78	0.58			0.17		Diploma	37	3.87	0.61			0.20		
2/<	49	3.91	0.60			0.34	0.21	Bachelor’s degree	40	3.78	0.60			0.06	0.15	
								Post-graduate	30	3.86	0.61			0.18	0.02	0.13
Total	150	3.80	0.62					Total	148	3.80	0.62					
Self-regulation																
None	25	3.24	0.76	0.18	0.36			Primary-Matric	41	3.41	0.68	0.44	0.43			
1	76	3.48	0.53			0.31		Diploma	37	3.52	0.52			0.15		
2/<	49	3.47	0.52			0.29	0.03	Bachelor’s degree	40	3.52	0.45			0.16	0.01	
								Post-graduate	30	3.33	0.59			0.13	0.32	0.33
Total	150	3.44	0.57					Total	148	3.45	0.57					

*Mean* Average, *SD* Standard deviation, *N* Number of participants, *3Y* 3-year-old children, *4Y* 4-year-old children, *5Y* 5-year-old children, *Dr KKDM* Dr Kenneth Kaunda District Municipality. The geographical location group of Dr Ruth Segomotsi was not tabulated in Table 3 due to the small representative sample of ≤ 2% in this group

Tables 1, 2 and 3 present the results of the percentile ranks, t-tests, and ANOVA analyses. The results are presented below, organised according to the DECA-P2 behavioural rating scale’s protective factors (initiative, self-regulation, and attachment/relationship) as well as behavioural concerns.

### Protective factors

The protective factors scale results can be described as ‘typical’, indicating that the children in the North West show a ‘typical’ level of resilience-related behaviour. Protective factors consist of subscales, ‘initiative’, ‘self-regulation’, and ‘attachment or relationship’. This scale offers a comprehensive measure of the child’s social and emotional competencies associated with resilience. It serves as the most reliable and valid overall indicator of strengths within the DECA-P2 assessment. By distilling the child’s social and emotional strengths related to resilience into a single numerical value, the protective factors scale proves particularly useful for outcome measurement and programme evaluation purposes. It is important to note that a child may receive a ‘typical’ rating on the protective factors scale, yet still be identified as having an ‘area of need’ in a specific protective factor scale [30]. The children of the participants showed typical strengths in protective factors, as indicated by their overall T-scores. Independent t-tests revealed small differences by ethnicity and gender, while one-way ANOVAs showed minor variations by age, geographical area, number of siblings, and parental education concerning protective factors. Minimal variations further support the interpretation that resilience was broadly evident across demographic categories.

#### Initiative

Children in the North West have ‘typical’ initiative. *Initiative* measures a child’s ability to use independent thought and action to meet their needs [30]. These children are often engaged learners who initiate or organise activities with peers, demonstrate strong problem-solving skills, exhibit responsibility, show self-awareness, and embrace challenges [30]. Based on the reported effect size, in addition to the independent t-tests, the effect size indicates (d = 0.38, p-value = 0.02) a medium effect. This shows that there is a medium difference in the means of the participants who have boys vs. those who have girls with regard to results on the initiative subscale. The participants with boys (mean = 3.69, SD = 0.61) on average chose occasionally to frequently on the statements, the participants who had girls (mean = 3.92, SD = 0.62) on average leaned towards selecting frequently on the statements. This reflects that, within this sample, girls displayed slightly higher initiative skills than boys. Based on the reported effect size (d = 0.35, p-value = 0.02), the difference in the means of the participants who are geographically situated in Dr Kenneth Kaunda district vs. those who are from the Ngaka Modiri Molema District with regard to the initiative subscale is leaning towards showing a medium difference. A small difference in ethnicity was found in African and Caucasian groups concerning initiative. During the ANOVA test, there were minor differences in the children’s age and initiative. However, based on the reported effect size (d = 0.35, p-value = 0.23), there was a medium difference in the means of the participants who were geographically situated in Dr Kenneth Kaunda district vs. those who were from the Ngaka Modiri Molema District with regard to the initiative. The participants located in the Dr Kenneth Kaunda district (mean = 3.90, SD = 0.56) on average chose frequently to very frequently on the statements, and the participants from the Ngaka Modiri Molema District (mean = 3.70, SD = 0.57) on average selected occasionally to frequently on the statements. This reflects that participants living in Dr Kenneth Kaunda district’s children may display slightly more skills as measured by the initiative subscale compared to the participants with children living in Ngaka Modiri Molema District. The ANOVA test also indicated a medium difference between the initiative subscale and the number of siblings a child has, with an effect size of d = 0.34 (p-value = 0.22). Participants with a child who has two or more siblings (mean = 3.91, SD = 0.60) selected an average of occasionally to frequently on the statements compared to the participants with no siblings (mean = 3.65, SD = 0.77) who selected occasionally to frequently on the statements. This reflects that skills associated with initiative may be greater in children with multiple siblings compared to a child who has one sibling. Parental education showed a small difference in the initiative subscale.

#### Self-regulation

Children of the sample surveyed in the North West had ‘typical’ ‘self-regulation’ skills, indicating that the children in this context showed ‘typical’ behaviour, thereby demonstrating resilience. ‘*Self-regulation’* refers to a child’s capacity to express emotions and manage behaviour in healthy ways. These children generally deal with frustration and negative emotions without exhibiting challenging behaviours. They tend to be patient, cooperative, respectful, and considerate of others [30]. The independent T-test indicated that there were small differences in ‘self-regulation’ in the child’s gender and ethnicity, reinforcing that most children functioned within typical ranges regardless of subgroup. The one-way ANOVA indicated that there were small differences among aspects relating to ‘self-regulation’ and the child’s age, geographical area, number of siblings, and parental education.

#### Attachment or relationship

From the overall t-tests, the participants’ ‘attachment or relationship’ was identified as an ‘area of need’, meaning that participants described few behaviours in their respective children associated with attachments and relationships. This identified area indicates that parents view their children’s attachment and relationships as domains where additional support could strengthen socio‑emotional well‑being. Children with low SEWB often exhibit challenges in establishing and sustaining positive relationships with peers and adults [30]. They may be less inclined to actively engage with others and struggle to elicit positive attention and responses. These children are typically perceived as indifferent, cynical, and frequently displaying a negative emotional state [30]. No notable difference in ethnicity was found in African and Caucasian groups concerning ‘attachment or relationship’. There were small differences in the children’s age, gender, geographical area, number of siblings, and ‘attachment or relationship’. There were notable differences between the means of the group of participants who had primary schooling up to a level of a national certificate (matric) and the group of participants with a diploma, bachelor’s, and postgraduate degrees. The effect size ranged from 0.36 to 0.60 (p-value = 0.01), which indicates a medium difference in the relationship between parental education and attachment or relationship. Parents with the educational level of a national certificate or lower (mean = 3.93, SD = 0.60) on average selected *occasionally* to *frequently* on statements, whereas the parents with a diploma to a postgraduate degree (mean ranges from 4.15 to 4.29 with the SD ranging from 0.36 to 0.60) on average selected *frequently* on statements. This reflects that parents’ perception of their child’s attachment may improve as the parental education levels improve.

### Behavioural concerns

The ‘behavioural concerns’ regarding the children can be described as ‘typical’. The behaviours measured by this scale are characteristic of children experiencing issues with aggression, withdrawal, attention, and emotional regulation [30]. While high scores on this measure may indicate the presence of emotional and behavioural challenges, it is important to recognise that some of these behaviours may be developmentally typical for younger preschoolers and toddlers aged 2–4 years [52]. For the t-test regarding ‘behavioural concerns’ (effect size d = 0.35, p-value = 0.02), there was a medium difference in boys versus girls. Participants who had boys reported ‘rarely’ to ‘occasionally’ on the statements, and participants with girls, on average, selected ‘rarely’ on the statements. This reflects that more behavioural concerns were reported in boys (mean = 2.6, SD = 0.49) compared to girls (mean = 1.99, SD = 0.42). No statistical difference in ethnicity was found in African and Caucasian groups regarding ‘behavioural concerns’. There was a small difference in the child age and behavioural concern, but differences were reported in effect size; participants in the Ngaka Modiri Molema District had a medium difference in behavioural concerns compared to participants with children in the Dr Kenneth Kaunda (d = 0.40, p-value = 0.10) and Bojanala Platinum Districts (d = 0.37, p-value = 0.10). Participants in the Ngaka Modiri Molema District (mean = 1.93, SD = 0.41) on average selected ‘rarely’ to ‘occasionally’ on the statements, while participants from the Dr Kenneth Kaunda district (mean = 2.14, SD = 0.5) and the Bojanala Platinum district (Mean = 2.09, SD = 0.43) selected ‘rarely’ on the statements. This reflects that children from the Ngaka Modiri Molema District have more behavioural concerns compared to the other districts. Parental education showed a small difference in behavioural concerns.

## Discussion

The results of this study should be interpreted within the contextual realities, where access to early childhood mental health services and high-quality ECD resources that support social-emotional learning remains limited.

Based on the overall T-scores, the study’s findings indicate that preschool children in the North West exhibit ‘typical’ levels of protective factors, with typical functioning reported by parents or caregivers. Protective factors are characteristics of individuals, families, or communities that foster well-being and reduce adverse outcomes, with specific factors such as ‘initiative’, ‘self-regulation’, and ‘attachment/relationships’ playing a key role in building resilience [30, 53]. The absence of clear strengths in these domains underscores the need for early promotive interventions, particularly in contexts where services are underdeveloped [54]. Similar national findings have been reported, where risks such as substance abuse, economic challenges, neglect, abuse, and violence often outweigh protective factors [19]. These typical scores in protective factors in the present sample align with evidence showing that high-quality social-emotional learning programmes enhance social-emotional competence [33], pointing to the need for sustained integration and accessibility of these programmes into early childhood education in the North West province. Children in South Africa could benefit from a universal intervention programme to shield them from risk factors [55, 56].

In the present study, based on the overall T-scores, ‘attachment or relationship’ was found to be an ‘area of concern’. When attachment and relationships are an ‘area of concern’, preschoolers may benefit from additional support to strengthen the secure emotional base needed for healthy regulation, social connection, and resilience [57, 58]. This indicates that the children of the participants show fewer behaviours linked to secure attachments and interpersonal connections, highlighting opportunities to strengthen social and emotional development. Children with low SEWB frequently encounter challenges in building and maintaining positive relationships with peers and adults, including family members. They may be disinclined to actively engage with others and struggle to elicit favourable attention and responses. These children are often perceived as detached, cynical, and prone to expressing a negative emotional state [30]. The demographic variables identified in the present study are consistent with evidence that structural constraints increase the likelihood of inconsistent caregiving and relational instability, thereby contributing to attachment and relationship difficulties [59].

While this study did not directly measure family functioning, the protective factor of ‘attachment/relationships’ is closely linked to family support. Gaspar, Gomez-Baya [60] and Merkaš, Perić [61] found that a functioning family, characterised by high-quality interactions and support, was identified as a protective factor in child SEWB. Likewise, Dehnel and Dalky [62] identified relational support as a key protective factor that increases resilience. Empowering children and families to cultivate resilience through social support networks may represent a viable approach to preventing and managing mental health challenges that may be inaccessible or unaffordable through other treatment modalities in low-and middle-income countries. The COVID-19 pandemic likely exacerbated these vulnerabilities, as increased reliance on technology reduced opportunities for face-to-face interaction and modelling of social behaviours [61, 63].

From the ANOVA and independent t-tests, demographic variables such as ethnicity, gender, age, geographic area, and number of siblings did not directly impact ‘attachment or relationship’ scores. However, parental education was associated with differences in children’s ‘attachment/relationships.’ Studies in Europe and Asia similarly show that higher parental education levels are linked to improved attachment and self-regulation, partly due to greater access to quality early childhood education and enriched home environments. A study conducted in the Netherlands revealed that lower levels of parental education adversely impacted attachment and relationship skills, but added that it also adversely impacted emotional regulation and behavioural challenges among children [64]. Similar results were found in a sample of Southern Chinese preschoolers, where a higher parent education level was associated with improved levels of self-regulation and attachment abilities in pre-schoolers [65]. Alexandersen and Zachrisson [66] further clarify this association by indicating that parents with higher educational levels, regardless of their income level, are more inclined to enrol their preschool-aged children in early developmental educational facilities with enhanced structural quality that was measured by developmental resources, staff competence, and stability. In these educational settings, children experienced increased closeness to peers and educators and reduced conflict, which fostered improved relationship skills with both peers and adults. This suggests that a child’s ‘attachment or relationship’ quality may be positively influenced by parental education levels. Children who can form secure, positive relationships with educators, caregivers, peers, and family members are more likely to regulate their emotions and behaviour [67–69]. In the present study, the association between parental education and ‘attachment or relationship’ outcomes also reflects broader patterns in which children from more educated households receive stronger emotional support, leading to improved ‘attachment or relationship’ skills. This suggests that strengthening parental education and support may indirectly enhance children’s SEWB, reinforcing the importance of parent-focused interventions in South Africa.

‘Self-regulation’ scores in this study were ‘typical’, indicating that the children in this study generally managed emotions and behaviours constructively. These children typically deal with frustration and negative feelings without exhibiting problematic conduct. Small differences in ‘self-regulation’ were observed across demographic factors, consistent with international findings. Protective factors that influenced children’s ‘self-regulation’ are closely tied to parental resources such as cognitive abilities, psychological well-being, and economic stability [70, 71]. Chen and Yeung [71] found that parental resources available before the pandemic predicted fewer mental health challenges in children with positive parental control, helping to minimise challenges. ‘Self-regulation’ in early childhood is also linked to caregivers’ verbal skills, self-control, and positive parenting techniques, while Claussen and Robinson [70] demonstrated that children from financially and socially advantaged families displayed stronger ‘self-regulation’ than those from disadvantaged contexts. Similarly, families in which parents exhibit social-emotional competence are more likely to foster ‘self-regulation’ in their children [72].

‘Initiative’ scores were also typical; however, gender and environmental factors like number of siblings and demographic area influenced scores on the ‘initiative’ subscale. ‘Initiative’ skills refer to independent reasoning and problem-solving skills, showing responsibility and initiating interactions. Girls in the study demonstrated higher ‘initiative’ skills compared to boys. Consistent with international studies reporting greater engagement and curiosity among girls [73–75]. Literature suggests that exposure to situations that require problem-solving skills improves creativity and initiative in preschool children. This may explain the higher ‘initiative’ ANOVA scores in children in urban areas and with multiple siblings, as the latter children may have greater exposure to a variety of situations. Kelly and Camden [76] support this theory by arguing that initiative is influenced by habitual memory, indicating that one learns from repeated exposure to different situations. Gender and environmental factors influenced both initiative and behavioural concerns, highlighting the role of everyday contexts in shaping resilience-related behaviours.

In the present study, ‘behavioural concerns’ were reportedly higher in boys and those living in rural areas. A study done amid the pandemic in India, a low-and middle-income country, identified similar behavioural concerns in boys, limited parent-child interaction, and increased reliance on electronic devices, which contribute to behavioural challenges [77]. These results reinforce the need for accessible, parent-led interventions that address behavioural concerns early, before they escalate into long-term challenges.

Finally, the findings resonate with Sustainable Development Goal (SDG) 3.4, which emphasises reducing premature mortality from non-communicable diseases through mental health promotion. By identifying ‘areas of concern’ in preschoolers’ SEWB, this study establishes a baseline for designing promotive programmes that strengthen protective factors and support positive behavioural development. Evidence-based parenting interventions, which have been shown to improve developmental and emotional outcomes internationally [78–81] could be adapted for the South African context to fill current service gaps.

The study had some limitations. The sample was drawn from diverse backgrounds, but information about the participants’ family functioning, which may be crucial in the context of resilience, was not obtained. The researcher recommends future research on the relationship between family engagement and SEWB of children in low-and middle-income countries who are exposed to adversity. The study was conducted during a unique period with social restrictions, shortly after the COVID-19 pandemic. This study used convenience sampling from eight schools in the North West province, with participants largely reflecting stable, middle-class families (married, educated, homeowners, majority Caucasian). As such, the findings should be interpreted as exploratory insights into resilience and promotive needs within this specific context, rather than generalisable conclusions about all preschool children in the province. The results highlight typical functioning and resilience in initiative and self-regulation, while attachment emerged as an area for targeted support. Future research should include more diverse and representative samples under normal social circumstances, particularly from disadvantaged and under-resourced communities, to provide a fuller picture of preschoolers’ social-emotional well-being across socio-economic contexts. The study’s behavioural rating scale captured the observed experience of children as narrated by parents. It could have caused reporting bias. While the DECA-P2 has strong psychometric properties with international application across diverse populations, its use in the South African context requires careful consideration of cultural and contextual differences. To strengthen its applicability, future research should focus on validating the DECA-P2 within South African populations, including testing for construct validity and developing culturally appropriate norms. Despite these limitations, the study provides a valuable baseline for future research in developing countries such as South Africa.

## Conclusions

The study’s findings indicate that preschool children in this setting generally functioned within typical ranges, showing resilience, while attachment and relationships emerged as a specific area where further support could enhance socio-emotional well-being. Challenges faced by children in South Africa necessitate a focus on enhancing their resilience. Determining the SEWB of preschoolers could provide a baseline for developing targeted promotional SEWB programmes. The initial utilisation of the DECAP-P2 behavioural rating scale in South Africa has established a baseline measurement for future research. Multiple variables, such as gender, geographic area, parental education, and the number of siblings, influence the SEWB of preschool children, with parental factors being particularly crucial. Findings among the study participants from the North West Province, South Africa, indicated limited attachment and relationship behaviours, pointing to areas where promotive support could enhance socio‑emotional well‑being. Given the limited availability of specialised paediatric mental health care services, a targeted SEWB programme for children with similar characteristics to those in this study should emphasise ‘attachment or relationship’ skills. Secure, positive, and consistent relationships with caregivers foster higher levels of social-emotional well-being. To address identified SEWB needs, future programmes should focus on: nurturing stronger connections with caregivers and educators, enhancing parental emotional support to improve responsiveness within families, and expanding equitable access to structured SEWB programmes in early learning centres and health care services. Proactive universal promotion programmes can strengthen protective factors and foster socio‑emotional competence among preschool children in a developing country like South Africa.

## Data Availability

All data generated or analysed during this study are included in this published article.

## Abbreviations

ANOVA: Analysis of Variance
COVID-19: Coronavirus disease 2019
DECA-P2: Devereux Early Childhood Assessment Preschool Program, 2nd Edition
ECD: Early Childhood Development
HREC: Health Research Ethics Committee
SDG: Sustainable Developmental Goal
SES: Socio-economic status
SEWB: Social-emotional well-being
SPSS: Statistical Packages for Social Science
UCDG: University Capacity Development Grant
UCDP: University Capacity Development Programme
WHO: World Health Organization
